# Serum Irisin and Oxytocin Levels as Predictors of Metabolic Parameters in Obese Children

**DOI:** 10.4274/jcrpe.3963

**Published:** 2017-06-01

**Authors:** Çiğdem Binay, Cem Paketçi, Savaş Güzel, Nedim Samancı

**Affiliations:** 1 Tekirdağ Çorlu State Hospital, Clinic of Pediatric Endocrinology, Tekirdağ, Turkey; 2 Namık Kemal University Faculty of Medicine, Department of Pediatrics, Tekirdağ, Turkey; 3 Namık Kemal University Faculty of Medicine, Department of Medical Biochemistry, Tekirdağ, Turkey

**Keywords:** Child obesity, irisin, oxytocin, fat mass

## Abstract

**Objective:**

Irisin and oxytocin can affect energy homeostasis and it has been suggested that they may play an important role in reducing obesity and diabetes. In this study, we aimed to determine the relationship between metabolic parameters (including irisin and oxytocin levels) and anthropometric parameters in obese children.

**Methods:**

Ninety obese children (mean age, 13.85±1.63 years) and 30 healthy controls (mean age, 14.32±1.58 years) were enrolled in this study. Anthropometric and laboratory parameters (glucose, insulin, lipid, oxytocin, and irisin levels) were analyzed. The serum irisin and oxytocin levels were measured by enzyme-linked immunosorbent assay. Bioelectrical impedance was used to determine body composition.

**Results:**

Irisin level was higher in the patients than in the controls (p=0.018), and this higher irisin level was correlated with increased systolic blood pressure, body mass index, waist/hip ratio, fat percentage, fat mass, glucose level, insulin level, and homeostasis model assessment of insulin resistance. Serum oxytocin level was significantly decreased in obese children compared to the controls (p=0.049). Also, among the 60 obese patients, oxytocin level was significantly lower in patients with than in those without metabolic syndrome (8.65±2.69 vs. 10.87±5.93 ng/L, respectively), while irisin levels were comparable (p=0.049 and p=0.104, respectively). There were no statistically significant relationships between oxytocin or irisin levels and lipid levels (p>0.05).

**Conclusion:**

Obese children had significantly higher irisin levels than the healthy controls. Additionally, this study shows for the first time that oxytocin level is significantly lower in obese compared with non-obese children and also lower in obese children with metabolic syndrome compared to those without.

## What is already known on this topic?

The relationships among obesity-associated metabolic disturbances, insulin sensitivity, and circulating irisin levels have been investigated in both rats and humans. Oxytocinergic neurons in the paraventricular nucleus of the hypothalamus transmit hypothalamic adiposity signals to the nucleus of the solitary tract, a brain area that integrates satiety signals from the gut and hypothalamus. These signals are recognized to play an important role in body weight regulation and metabolic homeostasis.

## What this study adds?

To the best of our knowledge, no study on childhood obesity has investigated the relationship between oxytocin levels and metabolic parameters in children.

## INTRODUCTION

Both adipose and muscle tissues have been in focus during the last few years, because they are recognized to play an important role in body weight regulation and metabolic homeostasis. Interest in brown adipose tissue which increases after cold exposure, hormonal stimulation, expression of key genetic regulatory factors, exercise, and expression of a mitochondrial protein called uncoupling protein 1 has also increased in recent years (1,2). Intracellular signalling pathways regulate transcriptional factors such as peroxisome proliferator-activated receptor-γ co-activator 1-α (PGC1-α) which induces energy expenditure (3).

Irisin is a novel muscle-secreted peptide that is proteolytically processed from the product of fibronectin type III domain-containing 5, a type I membrane protein (2,4). Irisin is regulated by PGC1-α and has been proposed to mediate the beneficial effects of exercise on metabolism, inducing adipocyte browning and thermogenesis by increasing uncoupling protein 1 levels. In this context, stimulation of brown adipose tissue cells by irisin exposure may be a therapeutic method of improving metabolic homeostasis (5).

The relationships among obesity-associated metabolic disturbances, insulin sensitivity, and circulating irisin levels have been investigated in both rats and humans (6,7,8). The research findings have suggested that irisin is secreted by muscle tissue as well as adipose tissue, and that irisin secretion from subcutaneous adipose tissue is more relevant than that from visceral adipose tissue (1). Moreover, in addition to its role in skeletal muscle and adipose tissues, irisin might play a role in neural pathways, because animal studies have shown that irisin is expressed in cerebellar Purkinje cells (9). Whether irisin is expressed and plays a role in other brain areas such as the hypothalamus in obese individuals remains unclear.

Hypothalamic neuropeptides regulate energy intake by affecting the feelings of hunger and satiety (10). Oxytocinergic neurons in the paraventricular nucleus of the hypothalamus transmit hypothalamic adiposity signals to the nucleus of the solitary tract, a brain area that integrates satiety signals from the gut and hypothalamus (11). It has been proposed that oxytocin is regulated by PGC1-α and that neuronal inactivation of both PGC1-α and oxytocin leads to impaired thermoregulation and increased food intake (11,12,13).

Few studies have been performed to determine the circulating oxytocin levels in obese adults (14,15,16,17). Moreover, to the best of our knowledge, no study on childhood obesity has investigated the relationship between oxytocin levels and metabolic parameters in children. The objectives of this study were to determine irisin and oxytocin levels in obese children and identify the associations of oxytocin and irisin levels with metabolic and anthropometric parameters in obese children.

## METHODS

This prospective study included 90 children and adolescents (41 boys, 49 girls) aged 10 to 18 years. Of these, 60 were referred to the pediatric endocrinology outpatient clinic because of excessive weight gain and constituted the study group. Thirty healthy age- and sex-matched children served as the control group. The exclusion criteria were presence of chronic or hereditary diseases, of endocrinologic disorders including syndromes associated with obesity, and a history of drug use. The pubertal stage was assessed according to the criteria described by Marshall and Tanner (18). All participants were evaluated to be in stage ≥2. A challenge in determining the prevalence of metabolic syndrome (MS) is the multiple definitions and criteria used to identify this condition. In response, the International Diabetes Federation (IDF) released the IDF Consensus Worldwide Definition of MS as a single, universally accepted tool. The IDF defines MS in children and adolescents as the presence of abdominal obesity (waist circumference ≥90^th^ percentile for age and sex) and the presence of two or more of the following clinical features: an elevated triglyceride level (≥1.7 mmol/L), a low high-density lipoprotein cholesterol (HDL-C) level (<1.03 mmol/L), high blood pressure (systolic blood pressure ≥130 mmHg and/or diastolic blood pressure ≥85 mmHg and/or a diagnosis of hypertension), and an elevated glucose level (≥5.6 mmol/L and/or a diagnosis of type 2 diabetes) (19). Written informed consent was obtained from the parents after being informed about the aim and procedures of the study. The study protocol was approved by the Clinical Research Committee of Namık Kemal University School of Medicine.

Height was measured while in a standing position without shoes using a wall-mounted stadiometer sensitive to the nearest 0.1 cm (Harpenden, Holtain, Crymych, UK). Weight was measured using a portable calibrated scale sensitive to the nearest 0.1 kg (SECA762; Voge&Hakle, Hamburg, Germany) with the subjects wearing light clothing. Body mass index (BMI) was calculated as weight (kg) divided by height (m)2. Height, weight, and BMI were expressed as standard deviation scores (SDS) using the updated growth reference percentiles for Turkish children and adolescents (20). The fat mass percentage was obtained via bioelectrical impedance using the BC-418MA Tanita Segmental Body Composition Analyzer (Tanita Europe BV, Hoofddrop, the Netherlands). Waist circumference was measured around the patient’s unclothed abdomen at the narrowest point between the rib cage and superior border of the iliac crest. Hip circumference was measured in patients wearing light clothing at the level of the widest diameter around the buttocks using a nonstretch tape (21). Blood pressure was measured using an automated sphygmomanometer. Elevated blood pressure (≥95^th^ percentile for height) was determined using tables provided by the Task Force Report (22).

In both the study and control groups, peripheral venous blood samples were collected after a 12-hour overnight fast. The serum samples were separated from the complete blood samples by centrifugation at 3000 rpm for 5 min. The serum samples were then stored at −86 ºC in the freezer until irisin and oxytocin analysis. Glucose, insulin, free thyroxine (fT_4_), thyroid-stimulating hormone, low-density lipoprotein cholesterol (LDL-C), HDL-C, triglycerides, total cholesterol (TC), and alanine aminotransferase levels were determined in these samples using enzymatic methods (Roche Modular DP Automatic Biochemical Analyzer; Roche Diagnostics, Indianapolis, IN, USA). The homeostasis model assessment of insulin resistance (HOMA-IR) was used to determine the presence of insulin resistance by employing the following formula: fasting glucose (mmol/L) × fasting insulin (IU/L)/22.5. The HOMA-IR cut-off values for insulin resistance were calculated as 5.22 in boys and 3.82 in girls (23).

Serum irisin and oxytocin analyses were performed by enzyme-linked immunosorbent assay (ELISA). Serum irisin level was measured using an ELISA kit from Hangzhou Eastbiopharm Co., Ltd. (Hangzhou, China). The irisin range of the assay was 0.5 to 300 ng/mL. Serum oxytocin level was measured using the Eastbiopharm ELISA kit, and oxytocin range of the assay was 0.1 to 450 ng/L. The intra- and interassay coefficients of variation were <7% for oxytocin and <10% for irisin.

### Statistical Analysis

The data were analyzed using SPSS 20.0 Statistical Software (IBM Corp., Armonk, NY, USA). Variables were expressed as means ± standard deviations, medians (maximum-minimum), percentages, and frequencies. All variables were assessed following the preconditional control for normality and homogeneity of variance (Shapiro-Wilk and Levene tests). The groups were compared using an independent t-test or the Mann-Whitney U-test when the precondition was not provided. Associations between two continuous variables were evaluated by Pearson’s or Spearman’s correlation coefficient analyses when the parametrical test precondition was not provided. Categorical data were analyzed using Fisher’s exact test and the chi-square test. If the expected frequency was lower than 20%, the Monte Carlo simulation method was used to include this frequency in the analysis. The sensitivity and specificity of the oxytocin and irisin levels were evaluated using receiver operating curves. Youden’s index was then used to determine the cut-off points from the curves. A p-value <0.05 or <0.01 was considered statistically significant.

## RESULTS

Ninety obese children/adolescents (mean age, 13.85±1.63 years) and 30 healthy controls (mean age, 14.32±1.58 years) were enrolled in this study. The characteristics and baseline laboratory values of the patients and control subjects are shown in Table 1. BMI, BMI SDS, waist circumference, hip size, waist/hip ratio, fat percentage, fat mass, fat-free mass (FFM), glucose, insulin, HOMA-IR, TC, triglycerides, LDL-C, irisin, systolic blood pressure, and diastolic blood pressure were significantly higher and the oxytocin level lower in the obese patients compared with the controls (Table 1). No significant difference in sex was observed between the two groups (p>0.05). There were significant differences in the presence of acanthosis and insulin resistance between the patient and control groups (p<0.01).

In the patient group, a higher irisin level was correlated with increased systolic blood pressure, weight, weight SDS, BMI, BMI SDS, waist size, hip size, waist/hip ratio, fat percentage, fat mass, glucose, insulin, and HOMA-IR. There were significant relationships for the irisin level with systolic blood pressure at a rate of 31.8%, weight SDS at a rate of 30.8%, BMI SDS at a rate of 34.5%, waist/hip ratio at a rate of 26.1%, fat percentage at a rate of 25.7%, fat mass at a rate of 26.3%, glucose at a rate of 22.9%, insulin at a rate of 21.4%, and HOMA-IR at a rate of 23.2%. The above figures represent the proportion of patients in whom a significant relationship was seen. Additionally, statistically significant relationships were found between oxytocin and waist size at a rate of 21.2%, waist/hip ratio at a rate of 23.6%, the fat percentage at a rate of 24.3%, and fat mass at a rate of 22.9%. Oxytocin level was inversely correlated with these parameters (Table 2). There were no statistically significant relationships between the oxytocin or irisin level and lipid levels (p>0.05).

Of the 60 patients with obesity, 31.7% (n=19) had MS. The oxytocin levels were significantly lower in patients with than in those without MS (8.65±2.69 vs. 10.87±5.93 ng/L, respectively), while the irisin levels were comparable in the two groups (p=0.049 and p=0.104, respectively).

**Figure 1:**
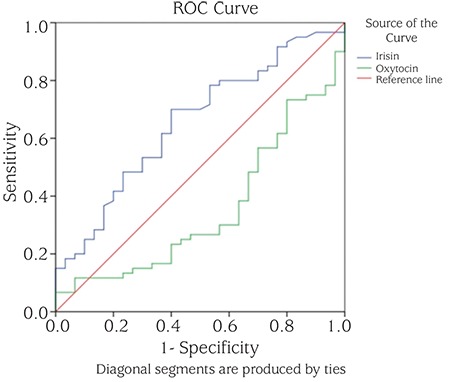
Receiver operating characteristic curve used to evaluate the sensitivity and specificity of serum irisin and oxytocin levels

The irisin level was found to be a statistically significant marker in discriminating the patients from the controls at a rate of 65.5%. The oxytocin level discriminated the patients from the controls at a rate of 35.4%. The cut-off points were 44.75 ng/mL for irisin, with a 70.0% sensitivity and 60.0% selectivity, and 8.30 ng/L for oxytocin, with a 56.7% sensitivity and 60.0% selectivity (Figure 1).

## DISCUSSION

Both adipose and muscle tissues secrete cytokines and other peptides such as adipokines and myokines, which are essential for metabolic homeostasis maintenance. Irisin and oxytocin were recently proposed to play important roles in reducing obesity and diabetes and improving life expectancy. Previously published results (7,24) have reinforced the concept that a correlation exists between irisin and BMI and suggest that irisin levels reflect the amount of adipose tissue in humans. Importantly, irisin was associated with an increased risk of MS and cardiometabolic variables in humans (25,26). The present study showed that increased irisin levels were correlated with a higher BMI, waist/hip ratio, fat percentage, fat mass, glucose level, insulin level, and HOMA-IR. These results are in agreement with recent reports in which a positive association was observed between circulating irisin levels and fasting insulin levels or HOMA-IR in studies on both adults (27,28) and children (29,30,31).

Crujeiras et al (32) investigated circulating irisin levels in a group of obese adults enrolled in a nutritional program to lose weight, after which some adults then gained weight. They demonstrated that irisin levels reflect net body adiposity. In addition, the authors later showed that irisin levels might predict the onset of insulin resistance in association with weight regain (27). Increased irisin levels have been proposed to serve as an adaptive response that compensates for the decreasing insulin sensitivity and metabolic disturbances associated with obesity (7,33). Irisin is increased in obesity in a manner similar to leptin, which suggests that irisin resistance develops similarly to that of leptin (27,34,35). On the other hand, it could be speculated that in obese individuals, a long-term increase in irisin promotes insulin secretion and insensitivity. Overall, the mechanism underlying increased irisin levels in obese patients remains unclear.

Al-Daghri et al (36) reported that in children, circulating irisin levels were correlated with impaired glucose tolerance and that this relationship was more evident in girls. Fasting blood glucose levels and HOMA-IR were negatively correlated, whereas BMI was positively correlated. However, in this study, the authors focused on sex differences and glucose metabolism rather than obesity and its metabolic consequences. The degree of obesity might be variable, because they found no correlation with BMI, in contrast to HOMA-IR. Palacios-González et al (29) reported that obese children had higher irisin levels which were positively correlated with BMI and leptin levels. Reinehr et al (30) analyzed the relationships among irisin, pubertal stage, obesity, and metabolic parameters in 40 obese children. They found that irisin levels were highest in obese children and were related to the pubertal stage as well as to many MS parameters, such as HOMA-IR, HDL-C, LDL-C, triglycerides, and diastolic blood pressure.

In contrast, some researchers have speculated that irisin levels are significantly decreased in patients with obesity (4), non-alcoholic fatty liver disease (37), and type 2 diabetes (38,39). This might be because of the various degrees of obesity among the patients included in the study groups. Although irisin is also secreted by adipose tissue, the reduced irisin levels in patients with type 2 diabetes may be a result of the decreased fat stores in patients with uncontrolled insulin deficiency, as is the case with leptin (27,40). Another study conducted in 65 obese children revealed no association of irisin levels with sex, age, pubertal stage, weight status, adipokines, or inflammatory markers (41). The different findings in our study may be attributed to the interventional design of the study. However, we did not evaluate the physical activity levels of our subjects.

Swick et al (35) found that irisin action can contribute to and account for differences among individuals whose energy expenditure was predicted by FFM, while FFM can explain approximately 80% of the 24-hour variance in energy expenditure. However, irisin levels were not correlated with the energy expenditure predicted by the FFM equation (35). Notably, in our study, an increased irisin level was correlated with a higher fat percentage and fat mass but was not correlated with FFM. Muscle tissue may be closely associated with changes in irisin levels after exercise training, whereas in pathologic situations such as obesity, adipose tissue is more closely associated with irisin regulation than are other tissues. Irisin showed a significant correlation with excess adiposity and slight correlation with the mass of other tissues (32).

Oxytocin is an anorexigenic neuropeptide that controls metabolic homeostasis not only via an effect on food intake but also by modulating energy expenditure (15). Few studies have been performed to determine the circulating levels of oxytocin in obese adults (14,15,16,17,42). Conflicting findings exist regarding whether oxytocin levels are increased (42), unchanged (43), or decreased (16,17) in obese adults. Variable sample sizes and different study techniques might be the causes of these differences.

In this study, we demonstrated for the first time that the serum oxytocin concentration was significantly decreased in obese children. Furthermore, oxytocin levels were significantly lower in obese children with than in those without MS; the irisin levels were comparable between these groups. Oxytocin level was significantly correlated with waist size, waist/hip ratio, fat percentage, and fat mass. In contrast, no relationship was detected between circulating oxytocin level and HOMA-IR. In our study, the HOMA-IR cut-off values for insulin resistance were 5.22 in boys and 3.82 in girls. However, one study suggested that the HOMA cut-off of 3.16 for insulin resistance is more reliable in adolescents (44). We might have produced different, significant results in our study if we used this cut-off level. We found no previous studies on oxytocin levels in obese children with which to compare our findings. According to our findings, we can state that decreased oxytocin levels may lead to impaired thermoregulation and increased food intake in obese children.

In their study on the influence of oxytocin in adults, Qian et al (16) reported that serum oxytocin levels were decreased in obese adults as well as in adults with type 2 diabetes. In that study, 176 subjects were enrolled, including 88 obese adults and 88 adults with type 2 diabetes; this sample was larger than our study group. In addition, the authors suggested that oxytocin might be involved in lipid metabolism because they found negative correlations with the TC, TG, and LDL-C levels (16). In contrast, we found no significant relationship between lipid levels and oxytocin or irisin levels. Very recently, Yuan et al (17) demonstrated that patients with MS had significantly lower oxytocin levels than did patients without MS, which is consistent with our findings. These reports suggest that pro-inflammatory cytokines may be a key factor in the ability of oxytocin to suppress the inflammation seen in MS.

The mechanism underlying decreased oxytocin levels in obesity remains unclear. Energy expenditure is regulated by many factors, including the transcriptional co-activator PGC-1α and intracellular signalling pathways. In animal studies, oxytocin has been shown to reduce food intake and induce fat weight loss (45,46). Animal studies have revealed that both PGC-1α knock-out and oxytocin receptor-deficient mice exhibit similar abnormalities and impaired thermoregulation and obesity (47,48). Blechman et al (49) showed that PGC-1α was necessary for production of the anorexigenic neuropeptide oxytocin in the zebrafish hypothalamus.

Potential novel clinical uses of oxytocin include treatment of diabetes, insulin resistance, obesity, and cardiovascular disease (50,51). Treatment with oxytocin was shown to reduce the expression of various pro-inflammatory cytokines (tumor necrosis factor-α, IL-1β, and IL-6) (52). These cytokines modulate insulin signalling responses in tissues (53). In addition, according to the results of another study, leptin modulates oxytocin levels and activates oxytocin neurons, thus leptin resistance may be overcome with oxytocin treatment (14).

One limitation of the current study is that our sample size was relatively limited; a larger cohort is needed to investigate this topic more thoroughly. Another limitation is that the physical activity status of the subjects was not evaluated. Finally, our findings did not address the underlying signalling mechanisms and associations between obesity and low oxytocin levels.

In conclusion, in this study, we found that circulating irisin and oxytocin levels were related to obesity in children. In addition, our results show for the first time that the oxytocin level is significantly decreased in obese children and in patients with MS. These findings remain to be confirmed and cannot yet be generalized to all patients. Larger study cohorts are needed to elucidate the mechanism underlying decreased oxytocin levels in obesity.

## Figures and Tables

**Table 1:**
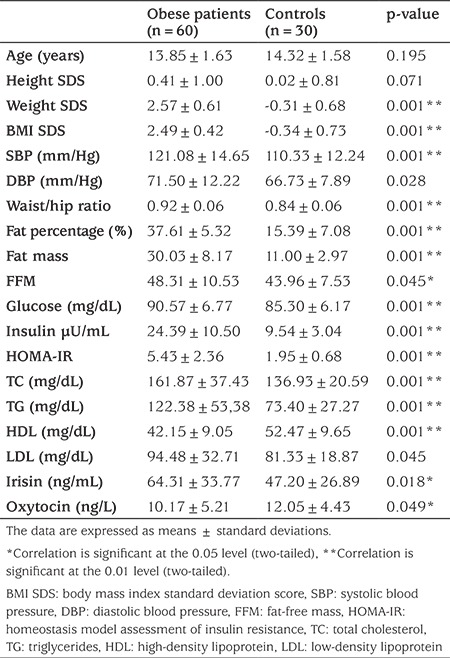
Baseline characteristics and metabolic parameters of the obese subjects and controls

**Table 2:**
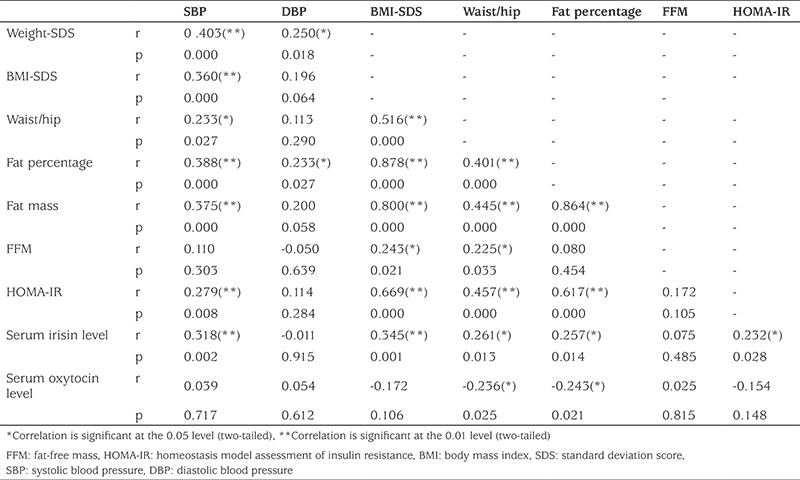
Correlation analysis of anthropometric and metabolic parameters
